# Repspiratory motion correction with a 2d self-navigator from bssfp dummy profiles

**DOI:** 10.1186/1532-429X-13-S1-P229

**Published:** 2011-02-02

**Authors:** Markus Henningsson, Peter Koken, Christian Stehning, Claudia Prieto, René M Botnar

**Affiliations:** 1 King's College London, London, UK; 2 Philips Research Europe, Hamburg, Germany

## Introduction

Several self-navigation techniques have been proposed to improve respiratory motion compensation in CMRA. Motion estimation is, however, often impeded by static structures within the excited volume. In this work a 2D self-navigation (2DSN) method is proposed and implemented by using the dummy profiles of a 3D bSSFP sequence. To create 2DSN images we added phase encoding (PE) gradients during the start-up echoes. With this approach we can calculate foot-head motion and retrospectively perform translational correction.

## Purpose

The objective of the study was to investigate the feasibility of respiratory motion correction using a novel self-navigation method and comparing it to a diaphragmatic 1D navigator (1Dnav).

## Material and methods

**Figure 1:**
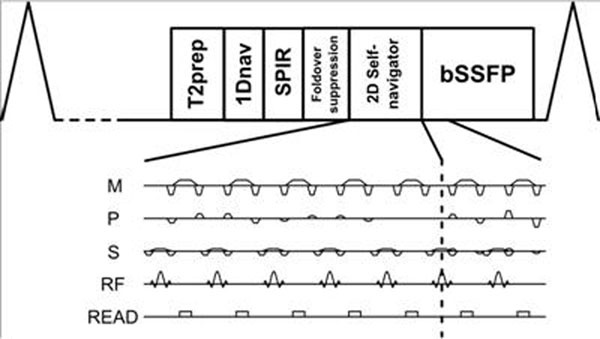
2D self-navigation coronary MRA sequence. Phase encoded dummy profiles are used for generation of navigator image.

**Figure 2:**
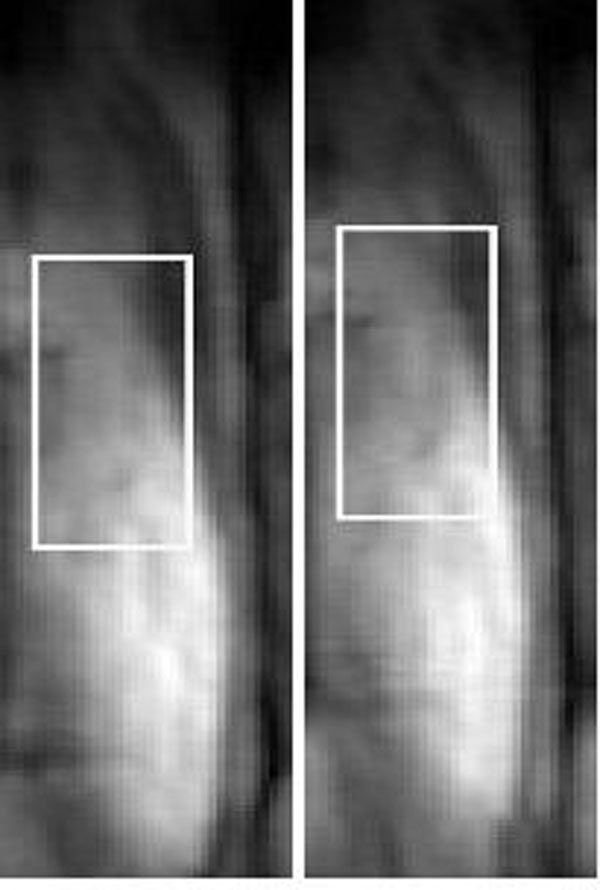
Two self-navigation images (sagittal view) at different respiratory phases. The white box illustrates the location of the tracked region-of-interest.

The proposed sequence is shown in Figure 1 where the number of dummy profiles corresponds to the 2DSN resolution in PE direction. No PE was performed in slice selection direction and the SNI are projections of the field-of-view in this direction. The 3D bSSFP imaging parameters included field-of-view=200x200x100mm, resolution=1x1x1mm, TE/TR=5.0/2.5ms, FA=70°. Ten profiles were acquired in each shot to create a 2DSN image with a resolution of 1x6x100mm. A template matching algorithm was used to extract motion from a region-of-interest, shown in Figure 2. A diaphragmatic 1Dnav was used for respiratory gating (10mm), and the 1Dnav values were also used for retrospective correction using a tracking factor of 0.6. Additionally, the data was reconstructed without motion correction. Two healthy volunteers were scanned on a Philips 1.5T Achieva scanner (Philips Healthcare, Best, NL) with the imaging plane in the sagittal orientation with PE in anterior-posterior direction and read-out in foot-head direction.

## Results

**Figure 3:**
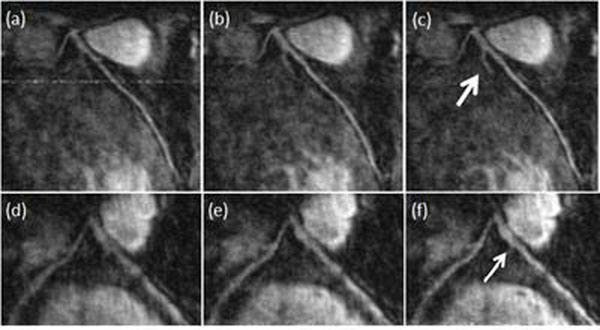
CMRA of LCA for two volunteers using: (a), (d) no motion correction (b), (e) 1D navigator with 0.6 tracking factor and (c), (f) self-navigating motion correction.

CMRA images of the left coronary artery without motion correction (a)(d), motion correction with the 1Dnav (b)(e), and using the 2DSN method (c)(f) are shown in Figure 3. The 2DSN approach visually improves the image quality compared to the 1Dnav (Fig 3c, f: arrows).

## Discussion and conclusion

The initial results in volunteers with the proposed self-navigation method are very promising as it provides a model-free motion correction method, which comes at no “expense” as the navigator is extracted from the dummy profiles used for magnetization preparation. Compared to other self-gating methods the main advantage of the proposed approach is that a spatial separation of the moving heart and static structures is possible. Future work will explore the use of motion correction with more degrees of freedom, e.g. affine motion, which is possible since the self-navigating method spatially encodes in 2D.

